# Impact of Dietary Feeding Levels of Juvenile Red-Tail Catfish (*Hemibagrus wyckioides*) in Land-Based Circular Tank: Insights From Metabolomics and Microbial Analysis

**DOI:** 10.1155/anu/5521491

**Published:** 2025-08-27

**Authors:** Baohong Xu, Haibin Hou, Tiaoyi Xiao, Changjun Chen, Qiaolin Liu

**Affiliations:** ^1^Fisheries College, Hunan Agricultural University, Changsha 410128, Hunan, China; ^2^Hunan Engineering Technology Research Center of Featured Aquatic Resources Utilization, Hunan Agricultural University, Changsha 410128, Hunan, China

**Keywords:** aquaculture industry, biochemical indices, China, feeding level, *Hemibagrus wyckioides*, land-based circular tank, metabolomics, microbiota, nutrition, quantitative real-time PCR

## Abstract

To investigate the most effective feeding level for red-tail catfish (*Hemibagrus wyckioides*) raised in land-based circular tanks and the impact of feeding levels on *H. wyckioides* at the molecular and omics levels, we conducted a 56-day experiment using the fish fries (16.49 ± 0.44 g). Three groups were established with varying feeding levels: 2% (T2), 3% (T3), and 4% (T4) of body weight per day. We compared conventional growth and physiological parameters, transcription levels of antioxidant activity, transforming growth factor (tgf), inflammatory factors, and lipid metabolism genes as well as intestinal microbiota and metabolomics. Our results showed that the feed conversion rate (FCR) in the T2 group was significantly lower than those in the other groups, but there were no significant differences in specific growth rate (SGR) and weight gain rate (WGR) among the groups. The T2 group had significantly higher level of albumin compared to the T4 group, while total protein (TP) and low-density lipoprotein cholesterol (LDL-C) levels were significantly lower than the T3 group. The T2 group also have significantly higher activities of superoxide dismutase (SOD) and alkaline phosphatase (ALP) compared to the other groups, and both alanine transaminase activity and goblet cells (GCs) were markedly elevated in the T2 group compared to the T3 group. Additionally, the T2 group had the lowest Firmicutes/Bacteroidetes ratio, with an increase in *Turicibacter* and *Clostridium*. The differential metabolites in the T2 group were significantly upregulated in amino acid metabolism and lipid metabolism-related pathways. The expression levels of antioxidant genes, tgf, anti-inflammatory factors, and lipid metabolism genes were all markedly elevated in the T2 group. These findings suggest that the optimal feeding level for *H. wyckioides* was 2% of body weight per day. These results can serve as a guide for the scientific aquaculture of *H. wyckioides*.

## 1. Introduction

The red-tail catfish (*Hemibagrus wyckioides*) is a large bagrid catfish species, known for its fast growth, large size, extensive adaptability, high disease and hypoxia resistance, lack of intermuscular bones, and high protein content. Due to these characteristics, it is considered an important economic species for aquaculture [1, 2]. Current research on *H. wyckioides* focuses on nutrition, genetics, breeding, and farming patterns [1–5]. For instance, as study found that adding 2.3% lysine to the diet of juvenile *H. wyckioides* significantly improved protein metabolism and antioxidant levels [3]. Another study showed that substituting 30% of fish meal with soybean meal in the diet of juvenile *H. wyckioides* led to significant improvements in growth performance [4]. However, there is still a lack of research on the optimal feeding level for *H. wyckioide*s, which hinders the improvement of breeding efficiency and economic benefits.

The feeding level has a significant impact on fish health, meat quality, and ultimately economic benefits. Appropriate feeding levels have been shown to greatly enhance the specific growth rate (SGR) and weight gain rate (WGR) of Chinese sturgeon (*Acipenser sinensis*) [6] and whiteleg shrimp (*Litopenaeus vannamei*) [7], and reduce the feed conversion rate (FCR) in juvenile snow trout (*Schizothorax zarudnyi*) [8]. Additionally, optimal feeding levels promote the activity of digestive enzymes [9].

The intestinal microbiota is closely associated with various host physiological processes, such as growth, development, and immunity [10]. It plays a crucial role in defending against invading pathogens [11]. Appropriate feeding levels significantly influence the intestinal microbiota, promoting intestinal health and growth in juvenile channel catfish (*Ictalurus punctatus*) [12] and blunt snout bream (*Megalobrama amblycephala*) [13]. Furthermore, feeding levels regulate the expression of nutritional metabolism and immune response genes in zebrafish (*Danio rerio*) [14].

Gut metabolomics can effectively reflect the host metabolic status, regulation mechanisms, and their association with signaling pathways [15]. For instance, Nie et al. [16] conducted an integrative analysis of the microbiome and metabolome, revealing that the gut microbiota substantially affects the growth of *Cyrinus carpio* through their unique metabolic functions in the rice-fish coculture system. Similarly, Zou et al. [17] used multiomics analysis to reveal differences in lipid metabolism in the gut between adult and juvenile yellowfin tuna (*Thunnus albacares*). They found that fatty acids, particularly short-chain fatty acids (butyrate and isobutyrate), glycerophospholipids, and sphingolipids, were significantly enriched in adults compared to juveniles.

To investigate the optimal feeding level for *H. wyckioides* farmed in land-based circular tanks and the mechanisms by which feeding levels affect the fish at molecular and omics perspectives, this study utilized a combination of physiological and biochemical indicators, gene expressions, microbiome compositions, and metabolomics analysis. The goal was to gain a comprehensive understanding of how feeding levels impact *H. wyckioides* and determine the optimal feeding level. This study applied multidisciplinary approaches, combining physiological, molecular, and omics analyses, to effectively bridge the gap in understanding how feeding regimens influence fish physiology, gut microbiota, and metabolic pathways. The findings offer practical insights for improving aquaculture efficiency.

## 2. Materials and Methods

### 2.1. Materials and Experimental Design

The experimental procedures were reviewed and approved by the Biomedical Research Ethics Committee of Hunan Agricultural University (Approval Number 2024165). The experiment was conducted at Yule Agriculture Co. Ltd. (Puer, China) using a land-based circular tank system. To ensure a sufficient number of experimental fish, 750 fish were provided by the company and acclimated for 2 weeks before being grouped. Then, 225 fish (*N* = 750, initial average weight: 16.49 ± 0.44 g) were randomly distributed into nine tanks with a diameter of 600 cm and a height of 150 cm (*n* = 25). The remaining fish were transferred to commercial ponds for commercial fish culture. The feed used in this experiment was purchased from Tongwei Co. Ltd. (Chengdu, China), and its composition included 36% crude protein, 4% crude fat, 15% crude ash, and 12% moisture. The fish were then divided into three feeding levels: 2% (T2), 3% (T3), and 4% (T4) of their body weight per day, with three feeding times per day. Each group had three tank replicates. The culture process lasted for 56 days and was maintained under a 12-h light–dark cycle. The water temperature ranged from 21 to 24.6°C, with a pH of 7.4 ± 0.2. The NH_4_-N concentration was less than 0.2 mg/L and the dissolved oxygen concentration was more than 5 mg/L.

### 2.2. Sample Collection

After the experiment, the fish were fasted for 24 h. Five fish were randomly selected from each tank and anesthetized though soaking them in a solution of 100 mg/L of tricaine methanesulfonate (MS-222; Sigma, USA) in a cuvette. The survival rate (SR), SGR, WGR, and FCR were calculated using methods previously reported [18, 19]. Blood samples were collected from the caudal vein and then centrifuged at 4000 rpm for 10 min at 4°C for subsequent serum biochemical analysis. Liver and intestinal tissues were collected in a sterile manner for gene expression analysis, whereas intestinal contents were collected for microbiota and metabolome analyses. These samples were then snap-frozen in liquid nitrogen and stored at −80°C. Intestinal tissues for histological sectioning were fixed in 4% formaldehyde.

### 2.3. Determination of Physiological and Biochemical Indicators

Condition factor (CF, g/cm^3^), hepatosomatic index (HSI, %), and viscerosomatic index (VSI, %) were calculated as described in a previous study [18]. The moisture, crude protein, crude fat, and ash contents were determined using standard methods [20].

Serum albumin, high-density lipoprotein cholesterol (HDL-C), low-density lipoprotein cholesterol (LDL-C), triglycerides (TGs), total cholesterol (T-CHO), glucose (GLU), total protein (TP), total antioxidant capacity (T-AOC), glutathione peroxidase (GSH-PX), malondialdehyde (MDA), superoxide dismutase (SOD), catalase (CAT), alanine aminotransferase (ALT), alkaline phosphatase (ALP), and protease (PR), amylase (AMS), and lipase (LPS) activities were measured using kits purchased from Nanjing Jiancheng Bioengineering Institute (Nanjing, China) [19].

### 2.4. Gut Histological Analysis

Gut histological examination was performed using hematoxylin and eosin (H&E) staining and observations were made under an optical microscope as a previously described [21].

### 2.5. Gene Expression

Total RNA was extracted using a total RNA extraction kit purchased from Nanjing Jianchen Bioengineering Institute (Jiangsu, China). Reverse transcription was performed using the PerfectStart Uni RT & qPCR Kit. The expression levels of the target genes were assessed using the 2^−*ΔΔ*CT^ method [22] with β-actin as the internal reference gene [19]. The targeted genes and primer sequences are provided in Table 1.

### 2.6. Intestinal Microbiota Analysis

Total microbial DNA was extracted from intestinal contents using the OMEGA Soil DNA Kit (Omega, Norcross, GA, USA). The V3–V4 region of the 16S rDNA gene was amplified with the primers 338F and 806R, as previously described [23]. The library was constructed using the TruSeq Nano DNA LT Library Prep Kit (Illumina, USA) and sequencing was carried out on an Illumina platform at Biotechnology Co. Ltd., Shanghai, China. Feature sequences (ASVs) were generated using QIIME2 2022.11 [24], and low-abundance ASVs were removed based on the Greengenes database. Beta diversity was calculated using Bray–Curtis distance matrices, followed by principal coordinate analysis (PCoA) for dimensionality reduction and visualization with the ggplot2 package of R software. Composition and abundance at the phylum and genus levels for each sample were analyzed and visualized in bar charts using QIIME2. Permutational multivariate analysis of variance (PERMANOVA) was applied to evaluate the differences in microbiota compositions among the groups [25]. Spearman correlation coefficients were calculated for the four ASVs, and SparCC analysis was applied to determine correlation coefficients (R values) based on the RMThreshold method using random matrix theory. A microbial co-occurrence network was constructed with a unified similarity threshold of 0.94. The metabolic functions of the intestinal microbiota were predicted using PICRUSt2 in the MetaCyc (https://metacyc.org/) and Kyoto Encyclopedia of Genes and Genomes (KEGG, https://www.kegg.jp/) databases.

### 2.7. Gut Metabolome Analysis

Gut metabolites were extracted using the traditional methanol–chloroform extraction method, with quality control (QC) samples used for internal QC. Metabolite quantification and identification were performed using a Thermo Scientific liquid chromatograph–mass spectrometer (LC-MS) platform, Compound Discoverer 3.3 version 3.3.2.31 (Thermo, Waltham, USA) and the MMS spectral library. Chromatography was carried out using an ACQUITY UPLC HSS T3 column (100 A, 1.8 µm, 2.1 mm × 100 mm). Mass spectrometry analysis was carried out using a Thermo Orbitrap Exploris 120 mass spectrometer, with data acquisition performed in data dependent acquisition (DDA) mode on Xcalibur software version 4.7 (Thermo, Waltham, USA).

The raw data in raw format were imported into Compound Discoverer 3.3 for peak extraction, alignment, and correction. The Fill Gaps algorithm was applied for missing peak imputation, followed by normalization based on the sum of peak areas. All detected metabolites were categorized according to KEGG and MetPA and identified using custom-built libraries as well as online databases including mzCloud (https://www.mzcloud.org/), LipidMAPS (https://www.lipidmaps.org/), HMDB (https://hmdb.ca/), MoNA (https://mona.fiehnlab.ucdavis.edu/), and the NIST_2020_MSMS spectral library. The MS1 mass tolerance was set at 15 ppm, while the MS2 match factor threshold was set to 50.

### 2.8. Statistical Analysis

Data were expressed as mean ± standard deviation. To assess normality and homogeneity of variances, the Shapiro–Wilk and Bartlett tests were performed using the lattice and MASS packages in R 3.4.2 [26]. One-way analysis of variance (ANOVA) with Tukey–Kramer post hoc test was conducted using SPSS 25.0. Differences with *p*  < 0.05 were considered significant.

## 3. Results

### 3.1. Changes of the Physiological and Biochemical Indicators

The FCR was significantly lower in the T2 group compared to the other groups (*p*=0.024; Table 2). However, there were no noticeable changes in the SGR, WGR, CF, SR, HIS, and VSI among the groups (*p* > 0.05; Table 2). The moisture level was significantly higher in the T2 group compared to the T3 and T4 groups (*p*=0.040; Table 2), whereas the crude fat content was significantly lower compared to the other two groups (*p*=0.001; Table 2). However, there were no noticeable changes in the crude protein and crude ash among the groups (*p* > 0.05; Table 2). The ALT activity in the T2 group was significantly lower than that of the T3 group (*p*=0.032; Table 2), while the activities of SOD and ALP were significantly higher in the T2 group compared to the T3 and T4 groups (*p* < 0.05, Table 2). Additionally, the T2 group exhibited the lowest TP and LDL-C levels, and the albumin level was significantly higher compared to the other groups (*p* < 0.05; Table 2). The HDL-C level was significantly lower in the T2 group compared to the other groups (*p*=0.046; Table 2), and the GLU and T-CHO levels were significantly lower in the T2 group compared to the T3 group (*p* < 0.05, Table 2). However, there were no noticeable changes in the T-AOC, MDA, CAT, and GSH-PX among the groups (p > 0.05; Table 2). The LPS activity was significantly higher in the T2 group compared to the other groups, while the PR activity was significantly higher in the T2 group compared to the T4 group (*p*=0.003; Table 2).

Gut histological observations revealed that the T2 group had a higher number of goblet cells (GCs) compared to the other groups (*p*=0.052; Table 2; Figure S1). However, there was no significant difference in the thickness of the muscle layer or the length of the villi among the groups (*p* ≥ 0.05; Table 2; Figure S1).

### 3.2. Gene Expression Differences

**Figure 1:**
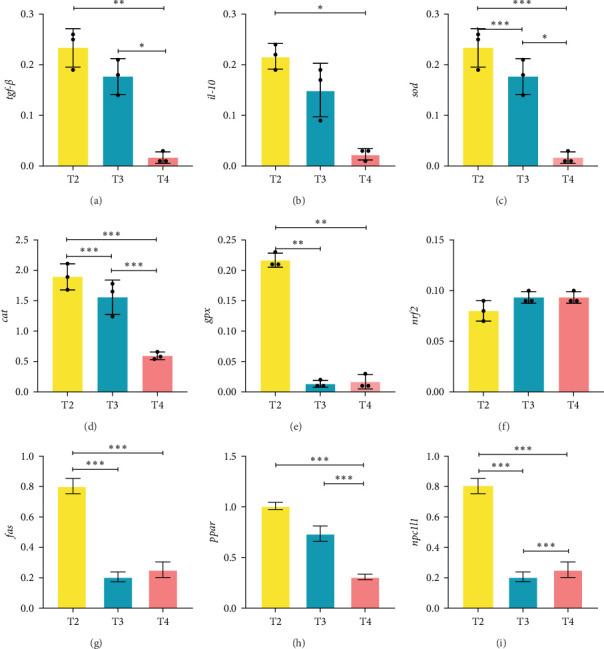
Effect of feeding levels on the mRNA expression levels of immune, antioxidant, and lipid metabolism genes in the liver of juvenile *Hemibagrus wyckioides*. (A) Transforming growth factor-β gene (*tgf-β*). (B) interleukin-10 gene (*il-10*). (C) superoxide dismutase gene (*sod*). (D) catalase gene (*cat*). (E) glutathione peroxidase gene (*gpx*). (F) nuclear factor erythroid 2-related factor 2 gene (*nrf2*). (G) factor-related apoptosis gene (*fas*). (H) peroxisome proliferator-activated receptor gene (*ppar*). (I) NPC1-like intracellular cholesterol transporter 1 gene (*npc1l1*). *⁣*^*∗*^*p*  < 0.05; *⁣*^*∗∗*^*p*  < 0.01; *⁣*^*∗∗∗*^*p*  < 0.001.

One individual was randomly collected from each tank for gene expression analysis, and therefore, each feeding level included three duplicate samples. The relative expression levels of *gpx* (Figure 1E), *sod* (Figure 1C), *cat* (Figure 1D), fatty acid synthase (*fas*; Figure 1G), and *npc1l1* (Figure 1I) genes were markedly elevated in the T2 group compared to the other groups (*p* < 0.05; Figure 1). The relative expression levels of interleukin-10 (*il-10*; Figure 1B), peroxisome proliferator-activated receptor (*ppar*; Figure 1H), and transforming growth factor-beta (*tgf-β*; Figure 1A) genes were significantly higher in the T2 group than in the T4 group (*p* < 0.05). The relative expression levels of *nrf2* gene were not significant difference among the groups (*p* ≥ 0.05; Figure 1F).

### 3.3. Differences of the Dominant Phyla and Genera in the Intestinal Microbiota

**Figure 2:**
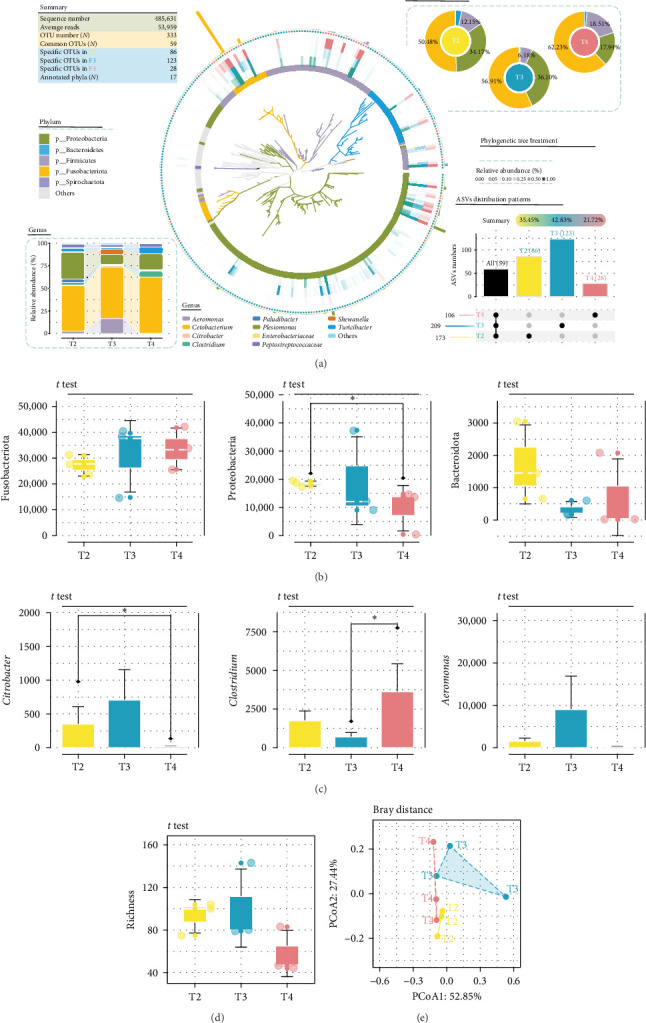
Effect of feeding levels on the intestinal microbiota in juvenile *Hemibagrus wyckioides*. (A) Relative abundances of dominant phyla. The upset plot shows numbers of unique and shared ASVs of each group. The histogram shows the composition of dominant genera in each group. The phylogenetic tree constructed using ggtree shows the distribution of ASVs in the phyla. (B) Significant variations in dominant phyla. (C) Significant variations in dominant genera. (D) Richness of the microbiota. (E) PCoA profile constructed based on Bray–Curtis distances. *⁣*^*∗*^*p*  < 0.05.

One individual was randomly collected from each tank for intestinal microbiota analysis, and therefore each feeding level included three duplicate samples. A total of 333 ASVs were identified, with significant differences in their distribution across the groups (*p* < 0.05; Figure 2A). Among these, 173 (35.45%), 209 (42.83%), and 106 (21.72%) ASVs were detected in the T2, T3, and T4 groups, respectively. Additionally, there were 59 (17.7%) shared ASVs across the three groups, with 86, 123, and 28 ASVs unique to the T2, T3, and T4 groups, respectively. These findings suggest that feeding levels significantly influenced the diversity of intestinal microbiota. The dominant phyla across all three groups were Firmicutes, Proteobacteria, Bacteroidetes, and Actinobacteria. However, the relative abundance of Firmicutes in the T2 group was significantly lower at 50.48% compared to the T3 (56.91%) and T4 (62.23%) groups (*p* < 0.05; Figure 2A). The relative abundance of Proteobacteria was similar in the T2 (34.17%) and T3 (36.10%) groups, but significantly lower in the T4 group (17.94%; *p*  < 0.05; Figure 2B). The relative abundance of Bacteroidetes was significantly higher in the T2 group compared to the other groups (*p* < 0.05). The predominant genera were *Aeromonas*, *Cetobacterium*, *Citrobacter*, *Clostridium*, *Paludibacter*, and *Plesiomonas*. Interestingly, the relative abundance of *Clostridium* was significantly higher in the T2 group compared to the T3 group, whereas the relative abundance of *Citrobacter* was significantly lower in the T2 group compared to the T3 group (*p* < 0.05, Figure 2C). No significant differences in richness indices were found among the groups (*p* > 0.05; Figure 2D). Furthermore, the relative abundance of *Vibrio* in the T2 group was significantly lower at 2.82% compared to the T3 group (16.56%; *p*  < 0.05). These results indicate that feeding levels significantly impacted the composition of intestinal microbiota (Figure 2E).

**Figure 3:**
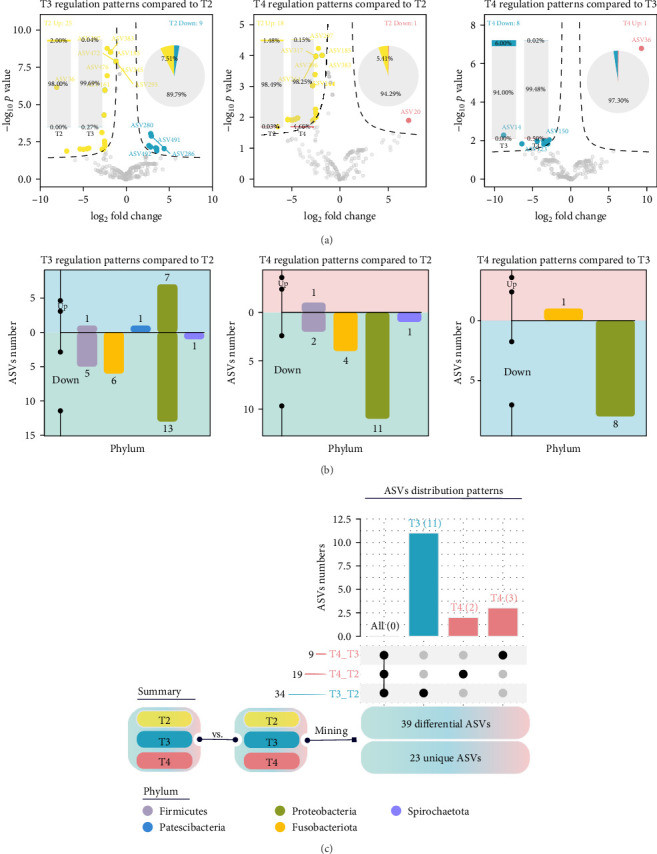
Differences in intestinal microbiota of juvenile *Hemibagrus wyckioides*. (A) Differences in ASVs between the T2 and T3 groups, between the T2 and T4 groups, and between the T3 and T4 groups. (B) Distribution of different ASVs in the phyla. (C) Distribution of differential ASVs.

**Figure 4:**
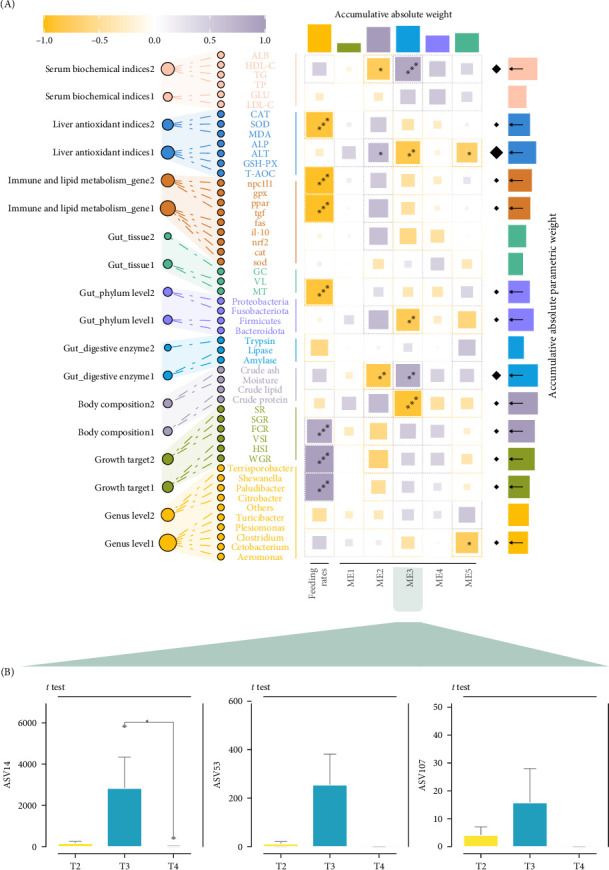
Potential regulatory patterns of juvenile *Hemibagrus wyckioides* parameters. (A) Correlation analysis based on Spearman coefficients, examining the interactions between module feature genes extracted from the abundance matrix of 23 differential ASVs and secondary module feature vectors derived from the phenotypic matrix. (B) Box plots show the abundances of identified biomarkers. *⁣*^*∗*^*p*  < 0.05; *⁣*^*∗∗*^*p* < 0.01; *⁣*^*∗∗∗*^*p* < 0.001.

Thirty-nine differential ASVs were detected among all samples (Figure 3C). Out of these, 23 ASVs were significantly influenced by feeding levels. In the T2 group, 25 ASVs were higher and nine ASVs were lower compared to the T3 group, whereas in comparison to the T4 group, 18 ASVs were higher and one ASV were lower (Figure 3A). The upregulated ASVs in the T2 group, when compared to the T3 group, mainly belonged to Proteobacteria and Firmicutes, while the downregulated ASVs primarily belonged to Firmicutes (Figure 3B). In comparison to the T4 group, the upregulated ASVs in the T2 group were predominantly from Proteobacteria and Actinobacteria, while the downregulated ASVs were mainly from Firmicutes (Figure 3B). Five module eigengenes (MEs) were identified and classified into a differential ASV abundance matrix, with phenotypic data organized into 16 characteristic genes (Figure 4A). The “serum biochemical indices2” and “body composition2” were significantly positively correlated with ME3, whereas “liver antioxidant indices1,” “gut phylum level1,” and “growth target T2 group” were significantly negatively correlated with ME3 (Figure 4A). Negative correlations were also observed between “liver antioxidant indices2,” “immune and lipid metabolism gene2,” “immune and lipid metabolism gene1,” and “gut tissue1.” On the other hand, “serum biochemical indices2” and “body composition2” were negatively correlated with ME2, whereas “liver antioxidant indices1” was positively correlated with ME2 (Figure 4A). The main contributors to ME2 and ME3 were three Proteobacteria microorganisms: ASV14, ASV53, and ASV107 (Figure 4B).

### 3.4. Impacts of Feeding Levels on Intestinal Microbiota

**Figure 5:**
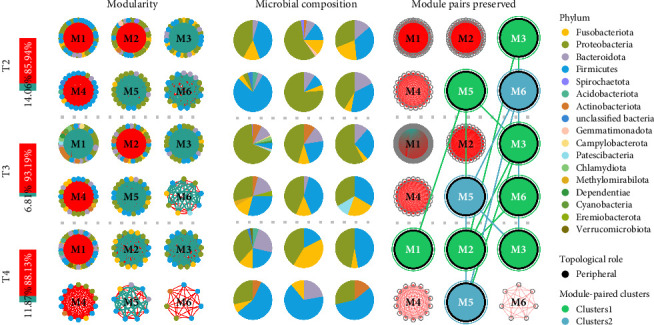
Modularity and ecological features of co-occurrence network of intestinal microbiota in juvenile *Hemibagrus wyckioides*. The similarity of network modules across groups was analyzed using Fisher's exact test. Similar module categories were grouped into module clusters and color-coded accordingly.

The neutral community modeling, based on the structural differences of the microbiota among the groups, revealed that as feeding levels increased, the fit of the microbiota in all groups decreased, indicating a significant influence of feeding levels on community structure. The Nm value in the T2 group was higher than that in the other groups, indicating a greater diversity of microbiota in the T2 group (Figure S2). At the phylum level, the molecular ecological networks of the groups showed high modularity, with the network divided into six submodules. The microbiota network in the T4 group was the most complex, with the highest number of nodes and edges, and the highest average degree (avgK), suggesting intense competition between the microbiota. A total of 108 module pairs were identified across the groups, with 18 single modules and 17 significantly different module pairs retained, accounting for 15.74%. These were clustered into two module clusters (Figure 5). The T4 group showed a loss of Cluster 3 modules, with only some modules assigned to Clusters 1 and 2, indicating that excessive feeding levels negatively impacted its microbiota structure and led to a loss of ecological function. The molecular ecological networks predominantly exhibited positive correlations, with the proportion of positive correlations ranging from 85.94% to 93.19%. Negative correlations were less common, with the T2 group showing the lowest proportion of negative correlations (6.81%), while the T4 group exhibited more negative interactions, which could potentially affect the stability and diversity of the microbiota.

### 3.5. Impacts of Feeding Levels on Gut Metabolome

**Figure 6:**
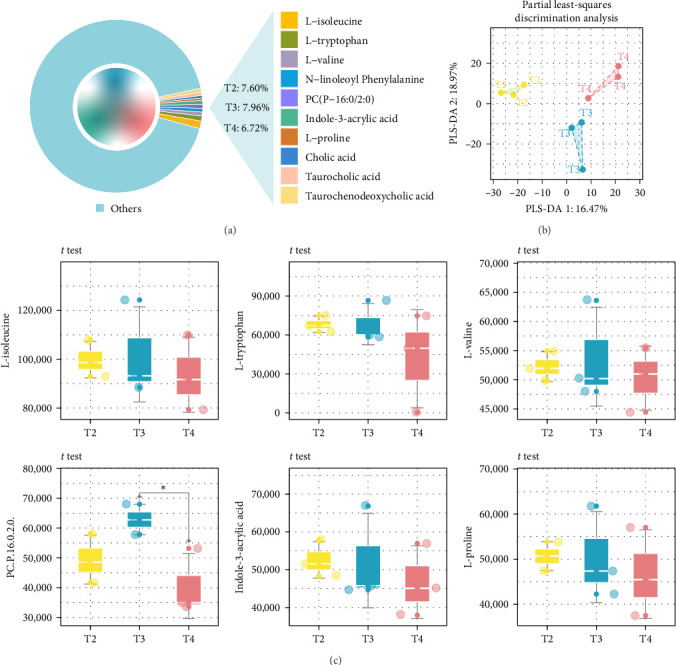
Compositions and differences of gut metabolites in juvenile *Hemibagrus wyckioides* under different feeding levels. (A) The major metabolites in the gut contents. (B) PLS-DA plot of the identified metabolites. (C) Changes of the primary metabolites. *⁣*^*∗*^*p*  < 0.05.

**Figure 7:**
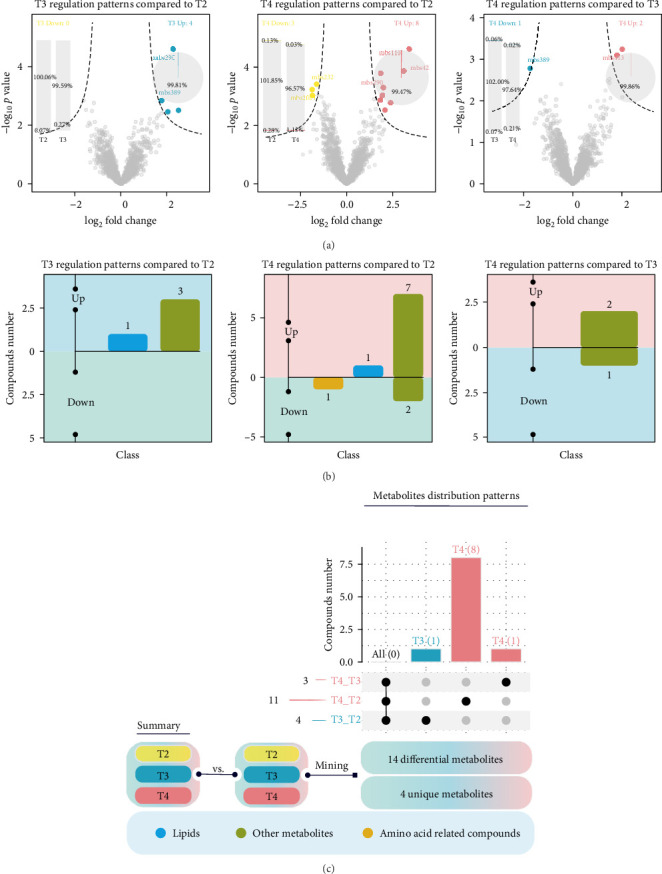
Differential metabolites (DMs) in the gut contents of juvenile *Hemibagrus wyckioides*. (A) Differences in metabolites between the T2 and T3 groups, between the T2 and T4 groups, and between the T3 and T4 groups. (B) Pairwise comparisons of DMs between different groups. (C) Comprehensive comparison of DMs among the groups.

One individual was randomly collected from each tank for gut metabolomic analysis, and therefore, each feeding level included three duplicate samples. The PLS-DA analysis revealed distinct clustering of the samples from the groups in the two-dimensional plot (Figure 6B), indicating that feeding levels significantly influenced gut metabolites. The main DMs included l-threonine, o-acetylserine, leucylproline, l-isoleucine, l-tryptophan, l-valine, l-proline, indole-3-acrylic acid, cholic acid, and taurodeoxycholic acid (Figure 6A). The abundances of D-PCP and l-valine were decreased in the T2 group compared to the T3 group, whereas the abundances of l-isoleucine, l-tryptophan, l-proline, D-PCP, and l-valine significantly increased compared to the T4 group (Figure 6C). The upset plot identified 14 DMs, with four metabolites in the T2 group showing changes compared to the T3 group (Figure 7A–C), primarily distributed in palmitoyl sphingomyelin and lipids. Eleven metabolites in the T2 group showed changes compared to the T4 group (Figure 7), with three upregulated and eight downregulated, mainly in palmitoyl sphingomyelin and lipids.

The DMs were annotated to seven KEGG secondary pathways and 30 tertiary pathways. The lowest abundance of tertiary metabolic pathways was 0.1%. In the T2 group, the histidine, pyrimidine, glutathione, and carbapenem biosynthesis pathways were upregulated compared to the T3 group. Conversely, the glycerophospholipid, secondary bile acid biosynthesis, taurine and hypotaurine metabolism, and purine metabolism pathways were downregulated. In the T2 group, the glycine, serine, and threonine metabolism, beta-alanine metabolism, and glutathione, arginine, histidine, and lysine metabolism pathways were upregulated compared to the T4 group. However, the secondary bile acid biosynthesis and tropane alkaloid metabolism pathways were downregulated (Figure S3).

## 4. Discussion

Optimal feeding levels are crucial for the production performance of *H. wyckioides*. Appropriate feeding levels promote the allocation of energy for growth, thereby enhancing growth performance of juvenile mandarin fish (*Siniperca scherzeri*) [27]. Increased feeding levels in yellow catfish (*Pelteobagrus fulvidraco*) [28] and Nile tilapia (*Oreochromis niloticus*) [29] have been shown to result in a corresponding increase in FCR, reduced feed efficiency, and higher farming costs. Our results showed that the FCR was significantly reduced in the T2 group, suggesting that feeding 2% of body weight per day was the most optimal for *H. wyckioides* farmed in the land-based cylindrical tanks.

Albumin has been shown to possess anti-inflammatory, antioxidant, and immune-enhancing properties [30]. In this study, the T2 group exhibited significantly higher level of albumin compared to the other groups, indicating a stronger immune system and disease resistance. SOD is a crucial indicator of antioxidant capacity and nonspecific immune function, playing a critical role in scavenging oxygen free radicals and reducing reactive oxygen species [31]. A decrease in SOD activity can lead to an increase in reactive oxygen species and MDA production, resulting in damage to the liver's antioxidant defense system [32] and triggering inflammatory responses [33]. Gut ALP acts as an antioxidant by detoxifying lipopolysaccharides and preventing inflammatory responses [34], while serum ALT is an important marker for assessing liver health, with elevated levels typically indicating liver damage or disease [35]. The T2 group showed the highest levels of SOD, ALP, and ALT activities, significantly higher than the other groups. These results suggest that the fish in the T2 group have better antioxidation and anti-inflammatory abilities compared to the other groups. However, it should be noted that the T2 group also faced a higher risk of the liver damage, although further verification is needed.

LPS and PR activity play a crucial role in the digestion and absorption of intestinal proteins and fats. Maintaining optimal levels of LPS and PR activity are essential for proper absorption of these nutrients. High feeding levels significantly reduced LPS activity, leading to inhibited fat digestion and absorption in red-finned pufferfish (*Takifugu rubripes*) [36]. Similar results were observed in research on gilthead seabream (*Sparus aurata*) [37] and giant river prawn (*Macrobrachium rosenbergii*) [38], where the activities LPS, PR, and AMS initially increased and then decreased with changing feeding levels, indicating a direct impact on the digestion and absorption of intestinal proteins and fats. In our study, the T2 group showed significantly higher LPS activity compared to the other groups, whereas the activities of PR and AMS followed a similar trend of initially increasing and then decreasing.

High feeding levels significantly reduced villus height, villus width, and muscle thickness, impairing the integrity of intestinal structure and inhibiting the growth of large yellow croaker (*Larimichthys crocea*) [9], juvenile golden pompano (*Trachinotus ovatus*) [39], and *I. punctatus* [40]. The presence of GC in the intestinal lining is crucial for secreting mucus and forming a protective mucosal layer, which helps to resist exogenous stimuli and pathogens [41]. In our study, there was no significant difference in the thickness of the intestinal muscle layer and the villus length among the groups. However, the T2 group showed significantly higher levels of GC compared to the other groups, indicating that a 2% feeding level in the T2 group may have strengthened the physical barrier of the mucosal layer, making it more effective in resisting exogenous stimuli and pathogens.

At low feeding levels, the abundance of *Firmicutes* in *I. punctatus* juveniles increased, leading to more active carbohydrate metabolism and enhanced energy and lipid metabolism [12]. The Firmicutes/Bacteroidetes ratio is an indicator of gut inflammation and microbial balance. Appropriate feeding levels can regulate this ratio and promote a healthy gut microbial ecology [42]. Our results showed that a low feeding level increased the abundance of Proteobacteria and reduced the Firmicutes/Bacteroidetes ratio, but did not change the abundance of Firmicutes. *Clostridium* species are probiotics that regulate intestinal inflammatory diseases and have significant anti-inflammatory effects [43]. They also improve growth, antioxidant activity, and gut health in juvenile grass turtles [44]. *Turicibacter* species can reduce serum T-CHO, TG, and adipose tissue mass, playing a crucial role in regulating host fat biology [45]. This genus greatly enhances growth, digestion, and gut health in yellow catfish (*Pelteobagrus fulvidraco*) [46]. In our study, the relative abundances of the beneficial *Clostridium* and *Turicibacter* were higher in the T2 group compared to the T3 group. *Vibrio* and *Citrobacter* are pathogenic genera that can cause diseases in the host [47]. Infections with *Citrobacter* in silver catfish (*Rhamdia quelen*) [48] and *A. sinensis* [49] can lead to severe bleeding in the liver and spleen, resulting in high mortality rates. Considering the dominant intestinal phyla and genera in relation to growth and health status, the T2 group showed better results than the other groups.

The intestinal microbiota plays a crucial role in regulating metabolic products and maintaining host health. This is achieved through the synthesis of active substances, degradation of amino acid, and production of coenzymes [50]. L-threonine, an essential component of mucin, is particularly important for gut homeostasis and immune regulation [51]. The production of L-glutamic acid and other short-chain fatty acids by *Bacteroides*, a type of intestinal microbiota, provides numerous benefits to the host by regulating intestinal metabolism and immune modulation [52]. L-glutamic acid also scavenges free radicals, alleviates oxidative stress, and enhances bacterial antioxidant enzyme activity [53]. O-acetylserine is another important substance produced by the intestinal microbiota, which helps maintain immune system homeostasis by stimulating glutathione synthesis and preventing autoimmunity [54]. Leucylproline, a dipeptide, plays a role in protein synthesis and cell signaling and is beneficial for promoting gut health and metabolism [55]. L-aspartic acid is an amino acid involved in protein synthesis, cell growth regulation, and gut energy metabolism [56]. Similarly, both l-valine [57] and l-isoleucine [58] are essential amino acids that play a crucial role in protein synthesis, energy metabolism, and inflammation regulation, thus, maintaining body growth and immune function. However, certain types of bacteria, such as *Pseudomonas* [59], *Lactobacillus* [60], and *Clostridium* [61], can cause harm to the host. In infected fish, these bacteria can lead to gut inflammation, hemorrhaging, and even mortality. In the T2 group, the abundance of beneficial *Bacteroides* was higher compared to the other groups and was positively correlated with l-glutamic acid and o-acetylserine. The abundance of *Clostridium* was higher in the T2 group than in the T3 group and was positive correlated with leucylproline and l-threonine. The abundance of pathogenic *Pseudomonas* was negatively correlated with l-aspartic acid and l-threonine, while *Lactobacillus* was positively correlated with D-PCP and l-valine, and negatively correlated with palmitoyl sphingomyelin. In summary, the abundance of beneficial *Clostridium* in the T2 group was higher than that in the T3 group, further demonstrating better intestinal homeostasis and immune regulation in the T2 group.

The metabolism of the gut microbiome is closely linked to the overall health of an organism and has a significant impact on growth [62]. For instance, alanine and glutamate play crucial roles in energy production, amino acid synthesis, and neurotransmitter regulation in amino acid metabolism [63]. Lipid metabolism is essential for providing aquatic animals with the necessary biochemical foundation to meet their nutritional requirements and enhance their immune capacity [64]. Overfeeding can lead to increased levels of TG and LDL-C, as well as elevated ALT activity, resulting in liver lipid deposition, reduced growth, and inflammation in *Larimichthys crocea* [33]. Feeding levels have been found to have a negatively correlation with PR activity, which can affect the digestion and metabolic efficiency of *Labeo rohita* [38]. Purine metabolism is crucial for RNA and DNA synthesis, signal transduction, metabolism, and energy homeostasis [65]. In this study, the DMs in the T2 group were primarily enriched in amino acid metabolism (such as glycine, serine, threonine, arginine, histidine, and lysine), lipid metabolism (including glycerophospholipid, primary and secondary bile acid biosynthesis), and purine metabolism compared to the other groups. This effectively confirms that maintaining an appropriate feeding level is beneficial for the intestinal metabolic function of *H. wyckioides*.

*tgf-β* is involved in cell growth and immune regulation and plays an essential role in various physiological and pathological conditions [66]. *il-10* is an anti-inflammatory cytokine that modulates immune cell activation [67]. The metabolite indole-3-acrylic acid promotes growth, development, and regulates inflammation [68]. High expression levels of both factors jointly regulate the growth and development of *H. wyckioides*. In the T2 group, the expression levels of *tgf-β* and *il-10* were significantly higher than those in the T4 group, indicating that the T2 group, with a lower feeding level, had superior intestinal anti-inflammatory and immune stress enhancement properties. *fas* is a metabolic oncogene and its aberrant expression is associated with various diseases, including cancer and obesity [69]. *ppar* is a nuclear receptor that controls gene expression, primarily involved in energy metabolism, cell development, and differentiation [70]. The protein encoded by the *npc1l1* regulates cholesterol absorption [71]. The metabolite cholic acid can promote lipid digestion and maintain intestinal and immune homeostasis [72]. The high expression of lipid metabolism genes and metabolites collectively suggests that *H. wyckioides* in the T2 group feeding level has a better lipid metabolism function. The gene expression levels of antioxidant genes (*sod*, *cat*, and *gpx*) and lipid metabolism genes (*fas*, *ppar*, and *npc1l1*) were also markedly elevated in the T2 group compared to the other groups. These findings suggest that appropriate feeding levels can promote growth and activate metabolic gene expressions through influencing intestinal microbiota and metabolic products of *H. wyckioides* farmed in a land-based aquaculture system.

It is important to acknowledge that there are potential limitations in this study. First, the short-term 56-day experiment may not accurately reflect long-term effects. Therefore, the parameters reported in this study may not show significant differences, but these differences may become apparent after a longer culture time. This should be further investigated through additional experiments. Second, this study only used juvenile *H. wyckioides*. It is necessary to conduct further studies to confirm whether the results are consistent in larger fish as well. Additionally, it would be beneficial to use special histochemical stains, such as Alcian blue-periodic acid-Shiff stain for GC counting [73] and oil red stain for adipocyte counting [74], to analyze specific cells in more detail. Unfortunately, this study did not focus on histological analysis.

## 5. Conclusion

The expression of growth and metabolic genes, compositions of intestinal microbiota, and metabolic products were all influenced by feeding levels, ultimately impacting the feed utilization rate and growth of *H. wyckioides*. Feeding 2% of body weight per day significantly improved the gut immunity, amino acid metabolism, and lipid metabolism functions in *H. wyckioides*. These findings suggest that the optimal feeding level for *H. wyckioides* in a land-based circular tank system was 2% of body weight per day with three feeding times per day. This discovery has the potential to improve the efficiency of *H. wyckioides* culture and reduce feed costs. Additionally, it may help alleviate pollution caused by *H. wyckioides* culture by reducing the amount of undigested nutrients from residual bait and feces released into the environment.

## Figures and Tables

**Table 1:** Primer sequences for real-time PCR of juvenile *Hemibagrus wyckioides*.

Gene	Nucleotide sequence (5′–3′)	Size (bp)	GenBank reference
Nuclear factor erythroid 2-related factor 2 gene (*nrf*2)	F: GCCTATGCTTACCCAGAATCCC R: GGCAGATACTGGCTGTAGTTGGA	162	XM_058392117.1
Superoxide dismutase gene (*sod*)	F: CGTGACCGCCAATTCCGATG R: CACCACAAGCCAGACGACCT	112	XM_058382267.1
Catalase gene (*cat*)	F: TCCGTCCTTCATCCACTCTCA R: GTCCATCAGGCAATCCACGAT	131	XM_058408555.1
Glutathione peroxidase gene (*gpx*)	F: GCATCACCTCGCTGTATCTCC R: TCGCCACATTCACAACCAGAG	168	XM_058382267.1
Interleukin-10 (*il-10*)	F: AAGCACCCATCGACACCATC R: TGCCTTTTCCTCCCATTTGAC	121	XM_058389472.1
Transforming growth factor-β gene (*tgf-β*)	F: CTTTGGACAGGAGAGGACGC R: TATTTTTACTGCCCGACTCCAC	115	XM_058391819.1
Factor-related apoptosis gene (*fas*)	F: CCTGAGTATGTGCGCAATGG R: ACAGACCACACTCCACAGAAC	98	XM_058399838.1
Peroxisome proliferator-activated receptor gene (*ppar*)	F: GCTGCGTACTGCGGTTTATG R: GGGCTAGGAAAGCGTCAAGT	124	XM_058373397.1
NPC1-like intracellular cholesterol transporter 1 gene (*npc1l1*)	F: ACGCATTCTGAGAGATGTACG R: CATCGTACTCAAAGCACCCAA	116	XM_058389472.1
*β-Actin*	F: AGAGGTATCCTGACCCTGAAGTAC R: GAGCATAACCTTCATAGATGGGCACAG	108	XM_058397995.1

**Table 2:** The impact of feeding levels on physiological and biochemical indicators of juvenile *Hemibagrus wyckioides*.

Parameters	T2	T3	T4	*p*-Value
FCR	1.349 ± 0.119^b^	1.497 ± 0.079^ab^	1.681 ± 0.115^a^	0.024
SGR (%/day)	1.199 ± 0.002	1.103 ± 0.001	1.224 ± 0.001	0.646
WGR (%)	52.922 ± 0.047	53.512 ± 0.008	54.756 ± 0.045	0.771
SR (%)	93.333 ± 0.083	96.000 ± 0.040	98.667 ± 0.023	0.531
HSI (%)	1.439 ± 0.003	1.307 ± 0.004	1.563 ± 0.003	0.296
VSI (%)	10.059 ± 0.020	10.145 ± 0.015	10.360 ± 0.020	0.939
CF (g/cm^3^)	0.009 ± 0.001	0.009 ± 0.001	0.009 ± 0.001	0.675
Moisture (%)	71.194 ± 0.006^a^	69.809 ± 0.004^b^	69.911 ± 0.007^b^	0.040
Crude protein (%)	17.832 ± 0.013	17.850 ± 0.003	17.870 ± 0.003	0.997
Crude fat (%)	5.994 ± 0.004^a^	7.978 ± 0.003^b^	8.061 ± 0.003^b^	0.001
Crude ash (%)	2.855 ± 0.003	2.779 ± 0.001	2.938 ± 0.001	0.233
TP (g/L)	27.468 ± 3.197^b^	35.214 ± 2.817^a^	25.609 ± 2.059^b^	0.002
Triglyceride (mmol/L)	7.412 ± 1.391^b^	10.747 ± 0.630^a^	7.669 ± 0.289^b^	0.007
HDL-C (mmol/L)	3.398 ± 0.345^b^	4.059 ± 0.654^ab^	4.360 ± 0.331^a^	0.046
LDL-C (mmol/L)	4.188 ± 0.854^b^	5.759 ± 1.295^a^	3.567 ± 0.833^b^	0.006
Glucose (mmol/L)	9.771 ± 2.207^b^	14.158 ± 0.945^a^	10.653 ± 2.444^b^	0.041
Albumin (g/L)	15.445 ± 1.171^a^	12.835 ± 0.923^ab^	12.546 ± 1.714^b^	0.045
T-CHO (mmol/L)	3.441 ± 0.715^b^	4.957 ± 0.717^a^	3.126 ± 0.585^b^	0.001
T-AOC (mmol/gprot)	0.305 ± 0.255	0.249 ± 0.134	0.309 ± 0.168	0.829
Malondialdehyde (nmol/mgprot)	0.945 ± 0.218	1.054 ± 0.251	0.977 ± 0.342	0.822
SOD (U/mgprot)	15.708 ± 0.136^a^	10.943 ± 0.792^b^	11.045 ± 0.791^b^	0.001
Catalase (U/mgprot)	69.332 ± 14.030	65.628 ± 6.513	59.975 ± 8.120	0.553
GSH-PX	60.306 ± 15.904	53.534 ± 4.318	70.153 ± 8.233	0.236
ALT (U/gprot)	14.120 ± 2.702^a^	8.495 ± 0.890^b^	11.747 ± 1.734^ab^	0.032
ALP (King U/gprot)	2.915 ± 1.605^a^	0.667 ± 0.423^b^	1.081 ± 0.548^b^	0.029
Protease (U/mgprot)	3.724 ± 0.872^a^	4.000 ± 0.853^a^	1.771 ± 0.157^b^	0.003
Amylase (U/mgprot)	1.010 ± 0.655^b^	3.942 ± 2.390^a^	1.053 ± 0.282^b^	0.046
Lipase (U/gprot)	52.878 ± 13.806^a^	29.808 ± 6.693^b^	31.403 ± 6.754^b^	0.009
GC (piece)	17.067 ± 9.573^a^	12.533 ± 5.431^b^	13.300 ± 7.193^ab^	0.052
MT (mm)	0.151 ± 0.048	0.141 ± 0.047	0.146 ± 0.031	0.668
VL (mm)	0.359 ± 0.129	0.306 ± 0.110	0.332 ± 0.095	0.198

*Note:* Values with different lowercase superscript letters in the same row indicate significant differences (one-way ANOVA, *p*  < 0.05). ALP, aspartate aminotransferase; MT, muscle layer thickness.

Abbreviations: ALT, alanine aminotransferase; CF, condition factor; FCR, feed conversion ratio; GC, goblet cell; GSH-PX, glutathione peroxidase; HDL-C, high-density lipoprotein cholesterol; HSI, hepatopancreas somatic index; LDL-C, low-density lipoprotein cholesterol; SGR, specific growth rate; SOD, superoxide dismutase; SR, survival rate; T-AOC, total antioxidant capacity of the liver; T-CHO, total cholesterol; TP, total protein; VL, villus length; VSI, viscera somatic index; WGR, weight gain ratio.

## Data Availability

The 16S rRNA sequencing data from this study have been uploaded to NCBI with accession number PRJNA1207803. The metabolomics data have been uploaded to Metabolights (www.ebi.ac.uk/metabolights/MTBLS12123). Other data will be available upon request.

## Supporting Information

Supporting Information Figure S1. Observation of gut tract morphology of juvenile *Hemibagrus wyckioides* at different feeding levels. (A–C) T2, T3, and T4, and (D–F) the local enlarged image of T2, T3, and T4. The black arrows represent goblet cells, the red lines represent villus height, and the black lines represent muscle thickness. Figure S2. Molecular ecological network analysis of the intestinal microbiota in juvenile *H. wyckioides*. (A) Evaluation of the community assembly process based on the neutral community model. A higher *R*^2^ indicates a better fit of the neutral model, suggesting a greater contribution of stochastic processes to community construction. (B) Circos plot for visualizing microbial interactions. The first row is based on microbial species, while the second row is based on abundance. The bands represent phylum, class, order, family, genus, and species from outside to inside. Red edges indicate positively correlated microbial interactions, and green edges indicate negatively correlated microbial interactions. Figure S3. KEGG pathway analysis of the metabolite.
