# Supplementary material for: Impact of Dietary Feeding Levels of Juvenile Red-Tail Catfish (Hemibagrus wyckioides) in Land-Based Circular Tank: Insights From Metabolomics and Microbial Analysis
Source: Aquac Nutr. 2025 Aug 27;2025:5521491. doi: 10.1155/anu/5521491 (PMC12408135; doi:10.1155/anu/5521491)

**Supplementary materials**

**Figure S1. Observation of gut tract morphology of juvenile *H. wyckioides* at different feeding levels.** A, B and C stand for T2, T3 and T4 respectively; D, E and F represent the local enlarged image of T2, T3 and T4, respectively. The black arrows represent goblet cells, the red lines represent villus height, and the black lines represent muscle thickness.


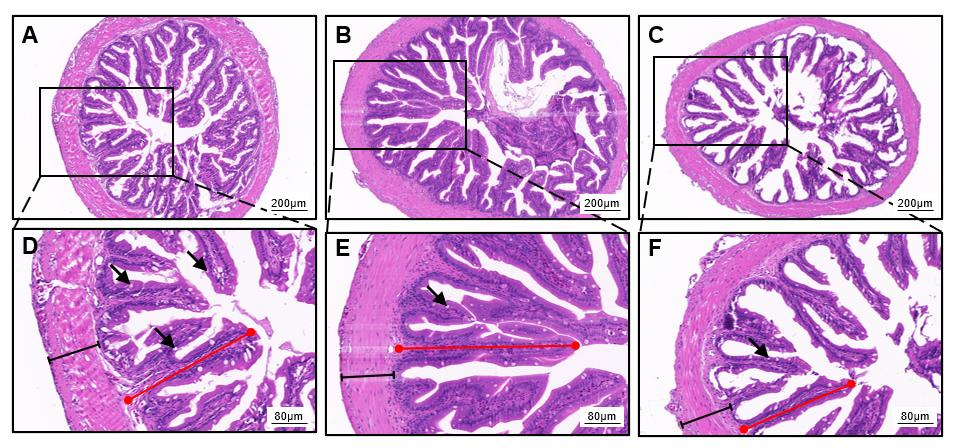


**Figure S2. Molecular ecological network analysis of the intestinal microbiota in juvenile *H. wyckioides*.** (A) Evaluation of the community assembly process based on the neutral community model. A higher R^2^ indicates a better fit of the neutral model, suggesting a greater contribution of stochastic processes to community construction. (B) Circos plot for visualizing microbial interactions. The first row is based on microbial species, while the second row is based on abundance. The bands represent phylum, class, order, family, genus, and species from outside to inside. Red edges indicate positively correlated microbial interactions, and green edges indicate negatively correlated microbial interactions.


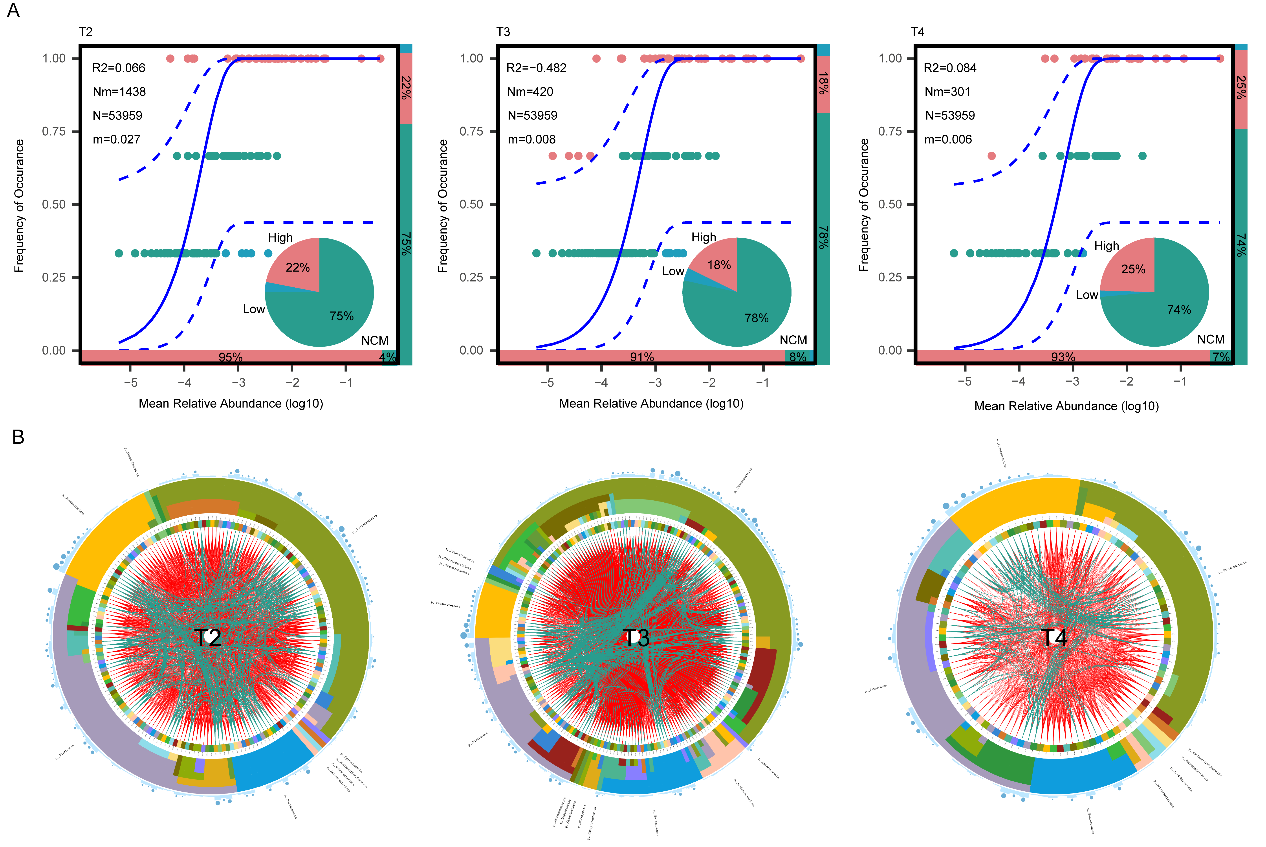


**F****igure S3. KEGG pathway analysis of the metabolite.**


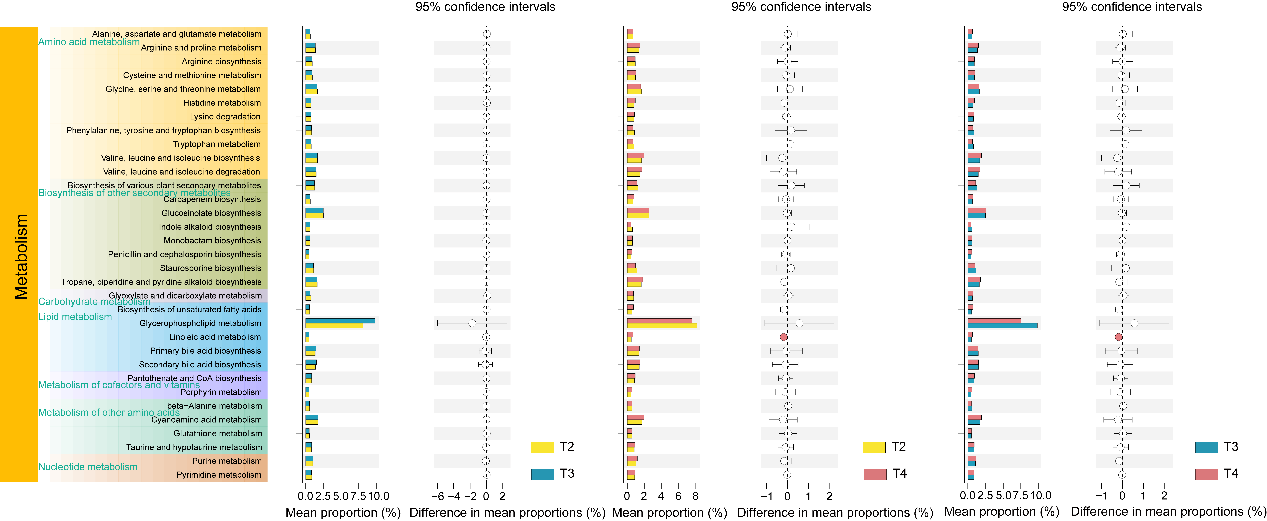

Supplement: Supporting Information — Figure S1. Observation of gut tract morphology of juvenile Hemibagrus wyckioides at different feeding levels. (A–C) T2, T3, and T4, and (D–F) the local enlarged image of T2, T3, and T4. The black arrows represent goblet cells, the red lines represent villus height, and the black lines represent muscle thickness. Figure S2. Molecular ecological network analysis of the intestinal microbiota in juvenile H. wyckioides. (A) Evaluation of the community assembly process based on the neutral community model. A higher R2 indicates a better fit of the neutral model, suggesting a greater contribution of stochastic processes to community construction. (B) Circos plot for visualizing microbial interactions. The first row is based on microbial species, while the second row is based on abundance. The bands represent phylum, class, order, family, genus, and species from outside to inside. Red edges indicate positively correlated microbial interactions, and green edges indicate negatively correlated microbial interactions. Figure S3. KEGG pathway analysis of the metabolite. [file 5521491.f1.docx]
